# Investigation on the Mechanisms of *Zanthoxylum bungeanum* for Treating Diabetes Mellitus Based on Network Pharmacology, Molecular Docking, and Experiment Verification

**DOI:** 10.1155/2023/9298728

**Published:** 2023-02-22

**Authors:** Yuanshe Huang, Zhaomiao Gong, Chen Yan, Ke Zheng, Lidan Zhang, Jing Li, E. Liang, Lai Zhang, Jingxin Mao

**Affiliations:** ^1^Anshun University, Guizhou Anshun 561000, China; ^2^Chongqing Medical and Pharmaceutical College, Chongqing 400030, China; ^3^An Shun City People's Hospital, Guizhou Anshun 561000, China; ^4^Department of Endocrine and Breast Surgery, The First Affiliated Hospital of Chongqing Medical University, No. 1 Youyi Road, Yuzhong District, Chongqing 400016, China; ^5^College of Pharmaceutical Sciences, Southwest University, Chongqing 400715, China

## Abstract

**Objective:**

The aim of the study was to explore the potential mechanism of *Zanthoxylum bungeanum* in the treatment of diabetes mellitus (DM) using network pharmacology.

**Methods:**

The DrugBank database and TCMSP platform were used to search for the main chemical components and their targets of *Zanthoxylum bungeanum*, and the genes related to diabetes mellitus were obtained from the genecards database. Import the data into the Venny 2.1.0 platform for intersection analysis to obtain the *Zanthoxylum bungeanum*-DM-gene dataset. The protein-protein interaction (PPI) analysis of *Zanthoxylum bungeanum*-DM gene was performed using the String data platform, and the visualization and network topology analysis were performed using Cytoscape 3.8.2. The KEGG pathway enrichment and biological process of GO enrichment analysis were carried out using the David platform. The active ingredients and key targets of *Zanthoxylum bungeanum* were molecularly docked to verify their biological activities by using Discovery Studio 2019 software. *Zanthoxylum bungeanum* was extracted and isolated by ethanol and dichloromethane. HepG2 cells were cultured, and cell viability assay was utilized to choose the suitable concentration of *Zanthoxylum bungeanum* extract (ZBE). The western blot assay was used for measuring the expression of AKT1, IL6, HSP90AA1, FOS, and JUN proteins in HepG2 cells.

**Results:**

A total of 5 main compounds, 339 targets, and 16656 disease genes were obtained and retrieved, respectively. A total of 187 common genes were screened, and 20 core genes were finally obtained after further screening. The antidiabetic active ingredients of *Zanthoxylum bungeanum* are kokusaginin, skimmianin, diosmetin, beta-sitosterol, and quercetin, respectively. The main targets for its antidiabetic effect are AKT1, IL6, HSP90AA1, FOS, and JUN, respectively. GO enrichment analysis revealed that the biological process of *Zanthoxylum bungeanum* and DM is related to a positive regulation of gene expression, positive regulation of transcription, positive regulation of transcription from RNA polymerase II promoter, response to drug, positive regulation of apoptotic process, and positive regulation of cell proliferation, etc. KEGG enrichment analysis revealed that common biological pathways mainly including the phospholipase D signaling pathway, MAPK signaling pathway, beta-alanine metabolism, estrogen signaling pathway, PPAR signaling pathway, and TNF signaling pathway. Molecular docking results showed that AKT1 with beta-sitosterol and quercetin, IL-6 with diosmetin and skimmianin, HSP90AA1 with diosmetin and quercetin, FOS with beta-sitosterol and quercetin, and JUN with beta-sitosterol and diosmetin have relatively strong binding activity, respectively. Experiment verification results showed that DM could be significantly improved by downregulating the expression of AKT1, IL6, HSP90AA1, FOS, and JUN proteins after being treated at concentrations of 20 *μ*mol/L and 40 *μ*mol/L of ZBE.

**Conclusion:**

The active components of *Zanthoxylum bungeanum* mainly including kokusaginin, skimmianin, diosmetin, beta-sitosterol, and quercetin. The therapeutic effect of *Zanthoxylum bungeanum* on DM may be achieved by downregulating core target genes including AKT1, IL6, HSP90AA1, FOS, and JUN, respectively. *Zanthoxylum bungeanum* is an effective drug in treatment of DM related to the above targets.

## 1. Introduction

Diabetes mellitus (DM) is one of the most serious endocrine disorders and noncommunicable diseases that threaten human health worldwide, mainly manifested as carbohydrate, fat, and protein metabolism disorders; the prevalence rate is increasing globally [1]. According to the International Diabetes Federation (IDF) global diabetes map in 2019, the number of diabetic patients was about 116.4 million people. Based on the IDF forecast, the number of diabetic patients in the world will reach 700 million in 2045, with more than 4 million people dying of diabetes and its complications, DM has become a very serious global health crisis [2]. According to research, DM is becoming a serious problem that threatens global health [3]. However, our current understanding of the cause and optimal treatment of this disease is not fully elaborated. The current drug treatment cannot control the continuous development of hyperglycemia well and even cause adverse reactions such as hypoglycemia, which will make patients bear further economic burdens. What is even more terrifying is the macrovascular and microvascular caused by hyperglycemia and hyperlipidemia. As well as damage to the heart, brain, and kidneys, the region's health system and economy are under enormous pressure [4]. Therefore, there is an urgent need to develop optimal and effective treatments and conduct in-depth research on the prevention and treatment of DM [5].

*Zanthoxylum bungeanum* is widely distributed in China, mainly in Sichuan, Guizhou, Gansu, Shaanxi, Chongqing, and other provinces. *Zanthoxylum bungeanum* is a traditional Chinese medicine (TCM) with a long history of edible and medicinal use [6]. It is rich in resources and has great development and utilization value. The 2020 edition of “Chinese Pharmacopoeia” recorded that *Zanthoxylum bungeanum* is warm in nature, pungent in taste, and returns to the spleen, stomach, and kidney meridians, and has the effects of warming and relieving pain, killing insects and relieving itching. The medicinal parts are the dried and ripe peel of the *Rutaceae* plant green pepper (*Zanthoxylum schinifolium* Sieb.et Zucc.) or Chinese *Zanthoxylum bungeanum* (*Zantohxylum bungeanum* Maxin.) [7]. The peel of *Zanthoxylum bungeanum* is rich in chemical components, mainly including volatile oils, alkaloids, amides, flavonoids, lignin, coumarin, triterpenoids, and other compounds [8]. The pharmacological activities of *Zanthoxylum bungeanum* are manifested in analgesia, anti-inflammatory, antioxidant, antibacterial, and antiobesity [9]. In recent years, many studies have been revealed that the extract of *Zanthoxylum bungeanum* exhibits the antidiabetic effect [10, 11].

Due to the complex components of TCM that takes a lot of manpower, material resources and time to study the molecular mechanism of TCM through animal experiments or cell experiments. In recent years, many scholars have studied the chemical constituents and pharmacological effects of *Zanthoxylum bungeanum*, but few studies have combined the chemical constituents and network pharmacology of *Zanthoxylum bungeanum*. With the development of bioinformatics and network pharmacology, technologies and methods based on biological data integration provide a convenient way to clarify the complex mechanism of action of TCM [12]. In the present study, the network pharmacology method was used to explore the mechanism of *Zanthoxylum bungeanum* in the treatment of DM. With the help of various public databases to obtain the effective active components of *Zanthoxylum bungeanum* and related antidiabetic targets, predict the antidiabetic targets of *Zanthoxylum bungeanum*, and conduct enrichment analysis for the relevant targets. We use Discovery Studio 2019 software for molecular docking to study the binding ability between receptors and small drug molecules and provide a reference for its in-depth research and clinical application. This study combined network pharmacology and molecular docking verification to explore the antidiabetic mechanism of *Zanthoxylum bungeanum*.

## 2. Materials and Methods

### 2.1. Collection of Active Components and Targets of *Zanthoxylum bungeanum*

All the chemical components of *Zanthoxylum bungeanum* were collected through the Traditional Chinese Medicine Systems Pharmacology Database and Analysis Platform (TCMSP), the threshold of oral bioavailability (OB) was ≥30%, and molecular weight (WM) was <500; the compounds were screened based on the drug-likeness (DL) threshold ≥ 0.18. After screening the main chemical components of *Zanthoxylum bungeanum*, the targets of active ingredients of *Zanthoxylum bungeanum* were screened out through the TCMSP analysis platform and DrugBank database.

### 2.2. Screening of Diabetes Mellitus Disease Targets

The GeneCards database provides comprehensive, user-friendly information on all annotated and predicted human genes. The DisGeNET database containing one of the largest publicly available collections of genes and variants associated with human diseases. Using the human GeneCards database (https://www.genecards.org/) and DisGeNET database (https://www.disgenet.org/) to search with “diabetes” OR “ diabetes mellitus” OR “DM” to obtain diabetes disease targets, respectively. The Venny 2.1.0 online mapping tool (https://bioinfogp.cnb.csic.es/tools/venny/index.html) was used to match the targets related to the active components of *Zanthoxylum bungeanum* obtained in Section 2.1 and the diabetes disease targets for mapping, draw a Venn diagram, and delete the duplicate targets to obtain the potential therapeutic targets of the active components of *Zanthoxylum bungeanum*.

### 2.3. Construction of Drug-Active Ingredient-Target Gene-Disease Network

The target genes corresponding to the active components of *Zanthoxylum bungeanum* obtained in Section 2.1, and the diabetes-related target genes screened in Section 2.2 were matched and mapped. The common genes of the two obtained were used as the key targets of *Zanthoxylum bungeanum* in the treatment of DM. The Cytoscape 3.8.2 software was applied to builds the relationship network of “drug-active ingredient-target gene-disease” and uses the “Analyze network” function in the software to perform topological analysis on the above network to clarify the mechanism of *Zanthoxylum bungeanum* in the treatment of DM.

### 2.4. Construction of Protein-Protein Interaction (PPI) Network of Diabetes Targets

The String database (https://cn.string-db.org/) was used to search for known and predicted PPI. Input the common targets of *Zanthoxylum bungeanum* and DM into the String database, select the study species as human (“Homosapiens”), set the confidence to be >0.4, hide disconnected nodes in the network, and use the downloaded.tsv file in String with R 4.0.4 language software is processed, and the PPI diagram is drawn according to the degree value (degree); the top 20 PPI core gene targets are finally obtained.

### 2.5. Gene Ontology (GO), Kyoto Encyclopedia of Genes, and Genomes (KEGG) Functional Enrichment Analysis

Based on the David database (https://david.ncifcrf.gov/), GO functional annotation and KEGG pathway enrichment analyses were performed on the intersection target genes of *Zanthoxylum bungeanum* and DM. The species was limited to “Human,” and the enriched entries with *q* values (that is, corrected *P* values) less than 0.05 were retained and sorted according to the size of the *q* values. The R 4.0.4 language software was applied to the obtained genes for GO and KEGG enrichment analyses and visualization. The GO analysis selects the top 10 enrichment results for display, and the KEGG analysis selects the top 20 enrichment results for display, respectively.

### 2.6. Component-Target Molecular Docking

The 2D structure of the active ingredient compound of *Zanthoxylum bungeanum* under item “1.1” was downloaded from the PubChem database (https://pubchem.ncbi.nlm.nih.gov/) and saved in “sdf” format as a small molecule ligand. We download the 3D structure of the protein corresponding to the core target from the RSCBPDB database (https://www.rcsb.org/) and save it in pdb format as a protein receptor. We use Discovery Studio 2019 software to process small molecule ligands, remove original ligands and water molecules, and add hydrogen atoms to the protein receptors to preprocess and find active pockets and then mix the core target protein receptors. Molecular docking with protein ligands [13] was done. The binding activity was evaluated by libdock score; the larger the libdock score, the better the binding activity and the more stable the docking. The Discovery Studio 2019 software was used to draw the binding mode diagram between the core target protein receptor and the small molecule ligand of the active ingredient of *Zanthoxylum bungeanum*, which was displayed in 3D and 2D structures, respectively.

### 2.7. Experiment Verification

#### 2.7.1. Reagents and Equipment

Fetal bovine serum and high sugar DMEM were purchased from Gibco Company. Trypsin, CCK8 kit, BCA protein concentration determination kit, rabbit anti-AKT1, IL6, HSP90AA1, FOS, and JUN were all purchased from Cell Signaling Technology and Beyotime Biotechnology, respectively. CO_2_ constant temperature incubator (Thermo Company), microplate reader (Tecan, Switzerland), electrophoresis apparatus (S1000, Biorad), electric blast drying oven (DHG-9070A, Shanghai Yiheng), gel imaging analysis system (Tanon-4600, Tianneng Technology), and SW-CJ-1FD/2FD ultra clean workbench (Jiangsu Suzhou Purification Equipment Factory) were used for the experiment.

#### 2.7.2. Material, Extraction, and Isolation

*Zanthoxylum bungeanum* (5 kg) was purchased from Jiangjin District, Chongqing, China, in May 2021. It was identified by Vice Professor Guowei Wang, College of Pharmacy, Southwest University of China. The voucher specimen (no. 20210015) has been deposited in the Herbarium of Anshun University. After being dried, *Zanthoxylum bungeanum* was extracted three times with 95% ethanol. The extract was evaporated to obtain a residue (0.5 kg), suspended in water, and fractionated with dichloromethane to obtain a *Zanthoxylum bungeanum* dichloromethane extract (ZBE, 54 g).

#### 2.7.3. Cell Experiment

*(1) Cell Culture*. Human hepatoma cell line HepG2 was purchased from the cell bank of Chinese Academy of Sciences. The frozen cells were quickly thawed, added to the culture medium, and centrifuged for 3 min; then, the supernatant was discarded, the cells were transferred to the culture bottle, and the prepared complete culture medium (10% fetal bovine serum, 1% penicillin streptomycin mixture, 89% high sugar DMEM) was added, and the cells were cultured in a 37°C, 5% CO_2_ incubator. When the cells in the culture bottle fuse to 80%~90%, they can be digested with trypsin and transferred to a new culture bottle.

*(2) Cell Grouping and Treatment*. Cell modeling was as follows: 38.5 mg of palmitic acid was dissolved in 1.5 mL of anhydrous ethanol to obtain a palmitic acid master batch at a concentration of 100 mmol/L. 0.5 mL of the master batch was diluted with high-sugar DMEM containing 1% BSA to obtain 10 mL of 5 mmol/L palmitic acid solution. After filtration and debacterization, the cells were incubated for 24 h with DMEM containing 35 mmol/L glucose to obtain 0.25 mmol/L of the modeling agent.

Grouping and drug administration was as follows: the control group (Ctrl) was given culture medium, the model group (Mod) and ZBE group were incubated in the incubator for 24 h after the intervention of the modeling agent by discarding the old medium and adding DMEM and ZBE extract with different concentration at 5, 10, 20, 40, and 80 *μ*mol/L, respectively.

*(3) Cytotoxicity and Cell Viability Assay*. The HepG2 cells were inoculated in 96-well plates at a density of 5 × 10^4^ cells/well in a volume of 100 *μ*L. After 24 h of cell culture, the cells were given a mold-making agent intervention for 24 h. The old solution was discarded, and the experimental groups were given different concentrations (5, 10, 20, 40, and 80 *μ*mol/L) of ZBE extract diluted in DMEM. After 24 h of administration, the medium was replaced with new medium; 10 *μ*L of CCK8 assay solution was added to each well, incubated in the incubator for 30 min, shaken, and mixed; and the optical density value (OD value) was read at a wavelength of 450 nm on an enzyme marker to calculate the cell growth rate.

*(4) Western Blot Assay*. The western blot method was used for AKT1, IL6, HSP90AA1, FOS, and JUN protein expressions in HepG2 cells. The HepG2 cells were inoculated in 6-well plates at a density of 2 × 10^5^ cells/well. After successful modeling, ZBE was added at 20 *μ*mol/L (low) and 40 *μ*mol/L (high), respectively, for 24 h. RIPA lysis solution was added to each well for 220 *μ*L, and the cells were lysed on ice for 10 min and scraped off and transferred to an EP tube, and the cells were repeatedly blown and broken in a cell crusher and continued to be lysed on ice for 10 min. The supernatant was extracted, and the protein concentration of each group was determined using the BCA kit, diluted and leveled using 5× protein loading buffer, and denatured in boiling water. The protein sample volume of 40 *μ*g per well was added to the precast gel, and electrophoresis buffer was added for electrophoresis (110 V, 80 min). The proteins were transferred to the PVDF membrane, blocked with 5% BSA for 1 h. The primary antibody (1 : 1000) was added overnight; the membrane was washed three times with TBST and incubated with goat anti-rabbit secondary antibody (1 : 1000) for 1 h. The membrane was washed three times with TBST and washed in ECL luminescent solution for color development and photographed, and the grayscale values of the bands were counted.

*(5) Statistical Analysis*. Statistical analysis was carried out using SPSS 20.0 software. The statistical significance of the differences was measured between groups by ANOVA, and all the data were statistically analyzed by mean ± S.D. *P* < 0.05 was considered statistically significant, and *P* < 0.01 was considered to be significantly different.

## 3. Results

### 3.1. Screening of Active Components of *Zanthoxylum bungeanum*

Taking OB, WM value, and DL properties as screening criteria, qualified compounds were screened from the TCMSP database. A total of 5 active components of *Zanthoxylum bungeanum* met the screening criteria, including kokusaginin, skimmianin, diosmetin, beta-sitosterol, and quercetin, respectively (Table 1).

### 3.2. Acquisition of Diabetes Target Extraction and Intersection Target

**Figure 1:**
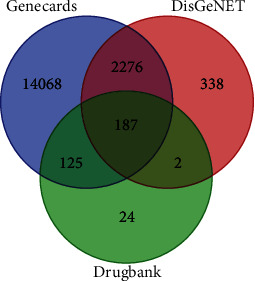
Venn diagram of the common target gene screening of *Zanthoxylum bungeanum* and diabetes.

Antidiabetes-related targets were obtained from GeneCards and DrugBank databases. A total of 339 drug component targets and 16656 antidiabetes-related targets were combined and intersected to obtain 312 *Zanthoxylum bungeanum* targets for the treatment of diabetes. The Venn diagram was drawn, as shown in Figure 1.

### 3.3. Construction of PPI Network and Screening of Core Targets

**Figure 2:**
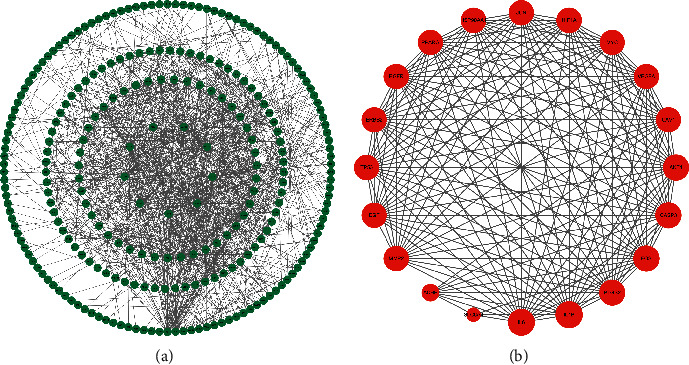
Construction of PPI network on *Zanthoxylum bungeanum* in the treatment of diabetes. (a) PPI visualization network diagram of 187 targets. (b) PPI visualization network diagram of 20 core targets.

A total of 312 common targets were imported into the string database. After removing duplicate and unverifiable targets, 187 targets remained to build the PPI network (Figure 2(a)). We import the obtained PPI network data into Cytoscape 3.8.2 software, analyze the network topology parameters, select the degree value greater than the median, and obtain 20 core targets (Figure 2(b), Table 2).

### 3.4. Construction of “Drug-Compound-Intersection Target” Interaction Network

**Figure 3:**
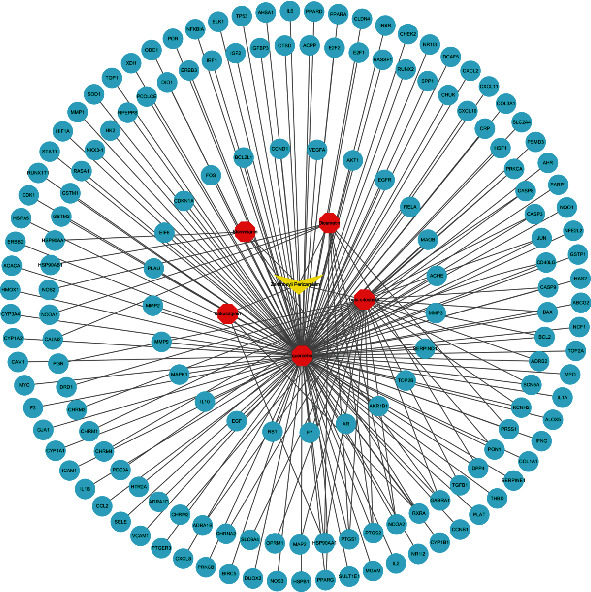
Construction of “drug-compound-intersection target” interaction network.

By comparing the degree values of each component in the “drug-compound -intersection target” network, the top 5 active components were selected including kokusaginin, skimmianin, diosmetin, beta-sitosterol, and quercetin, which could interact with 2, 4, 9, 31, and 145 target proteins, respectively. The top 5 target proteins were AKT1, IL6, HSP90AA1, FOS, and JUN (Figure 3).

### 3.5. GO Analysis Results of the Intersection Targets of *Zanthoxylum bungeanum* in the Treatment of DM

**Figure 4:**
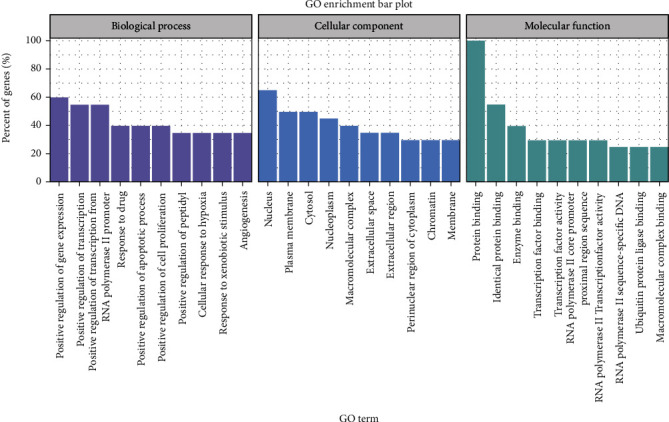
Bar diagram of GO functional enrichment analysis.

The R 4.0.4 software was used to conduct GO enrichment analysis on the 187 intersection targets of *Zanthoxylum bungeanum* in the treatment of DM. A total of 215 biological process items, 29 cell cellular items, and 39 molecular function items were obtained (all the results satisfy *P* < 0.05 and *Q* < 0.05), and the top 10 entries by Q-Value were visualized separately (Figure 4). The results of GO analysis showed that the biological pathways of *Zanthoxylum bungeanum*-diabetic disease intersection targets mainly including positive regulation of gene expression, positive regulation of transcription, positive regulation of transcription from RNA polymerase II promoter, response to drug, positive regulation of apoptotic process, positive regulation of cell proliferation, positive regulation of peptidyl, cellular response to hypoxia, response to xenobiotic stimulus, angiogenesis, nucleus, plasma membrane, cytosol, nucleoplasm, macromolecular complex, extracellular space, extracellular region, perinuclear region of cytoplasm, chromatin, membrane, protein binding, identical protein binding, enzyme binding, transcription factor binding, transcription factor activity, RNA polymerase II core promoter proximal region sequence, RNA polymerase II transcription factor activity, RNA polymerase II sequence-specific DNA, ubiquitin protein ligase binding, and macromolecular complex binding.

### 3.6. KEGG Metabolic Pathway Enrichment Analysis Results

**Figure 5:**
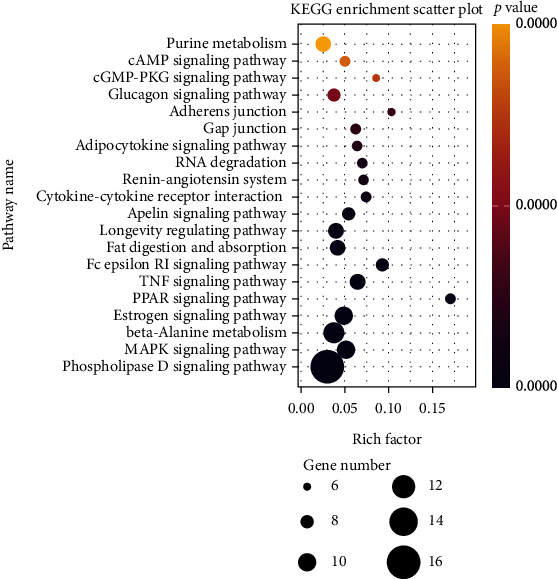
Bubble diagram of KEGG pathway enrichment analysis.

The KEGG pathway enrichment analysis of 187 intersection targets of *Zanthoxylum bungeanum* in the treatment of DM showed that there were 99 signaling pathways involved in the treatment of DM by *Zanthoxylum bungeanum* with a *P* value of less than 0.05, mainly enriched in the phospholipase D signaling pathway, MAPK signaling pathway, beta-alanine metabolism, estrogen signaling pathway, PPAR signaling pathway, TNF signaling pathway, fc epsilon RI signaling pathway, fat digestion and absorption, longevity regulating pathway, apelin signaling pathway, cytokine-cytokine receptor interaction, renin-angiotensin system, RNA degradation, adipocytokine signaling pathway, gap junction, adherens junction, glucagon signaling pathway, cGMP-PKG signaling pathway, and cAMP signaling pathway and purine metabolism. We take 20 pathways with significant differences and visualized them as bubble charts (Figure 5). The *P* value represents the significance of the enriched target. The smaller the *P* value, the redder the color, and vice versa. It reflects that the same target of TCM may participate in different biological processes, and the purpose of treating diseases can be achieved by improving biological processes.

### 3.7. Results of Molecular Docking

**Figure 6:**
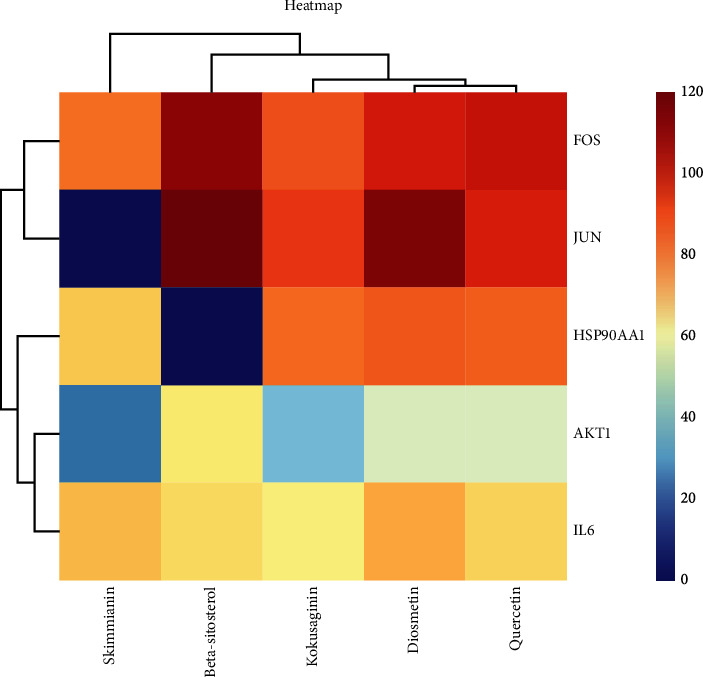
Heatmap of main active ingredients of *Zanthoxylum bungeanum* with 5 core target genes.

**Figure 7:**
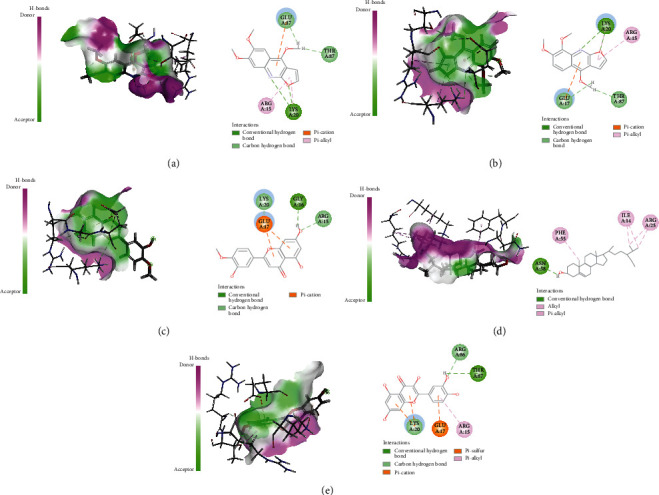
Molecular docking of AKT1 with 5 main ingredients: (a) kokusaginin, (b) skimmianin, (c) diosmetin, (d) beta-sitosterol, and (e) quercetin, respectively.

**Figure 8:**
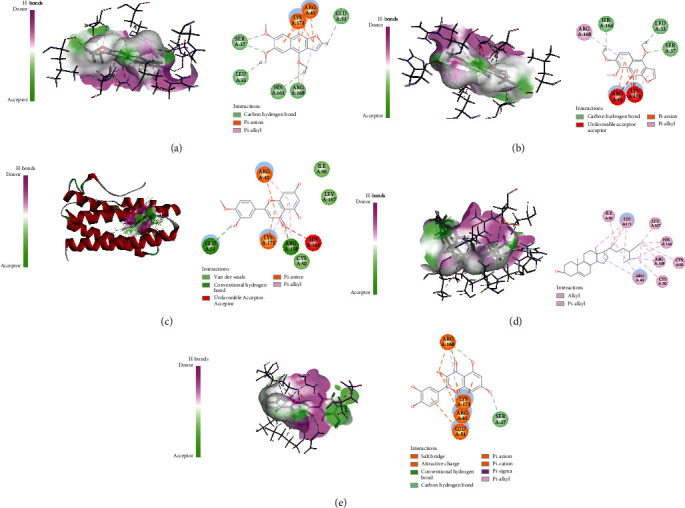
Molecular docking of IL-6 with 5 main ingredients: (a) kokusaginin, (b) skimmianin, (c) diosmetin, (d) beta-sitosterol, and (e) quercetin, respectively.

**Figure 9:**
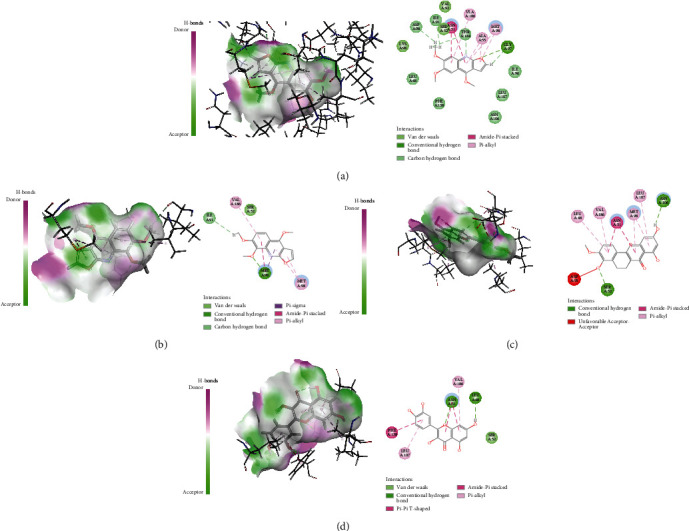
Molecular docking of HSP90AA1 with 5 main ingredients: (a) kokusaginin, (b) skimmianin, (c) diosmetin, and (d) quercetin, respectively.

**Figure 10:**
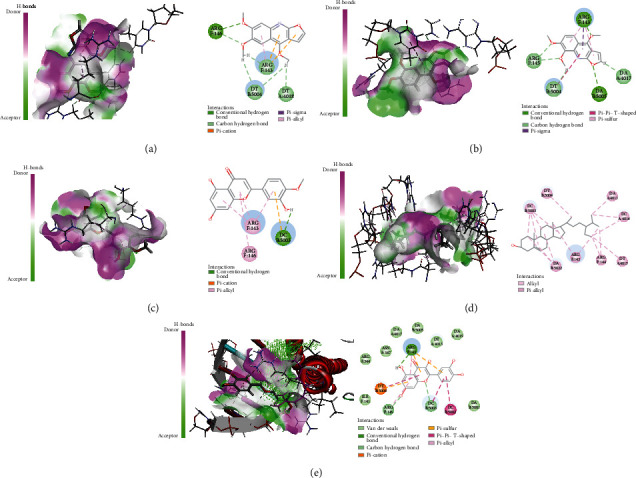
Molecular docking of FOS with 5 main ingredients: (a) kokusaginin, (b) skimmianin, (c) diosmetin, (d) beta-sitosterol, and (e) quercetin, respectively.

**Figure 11:**
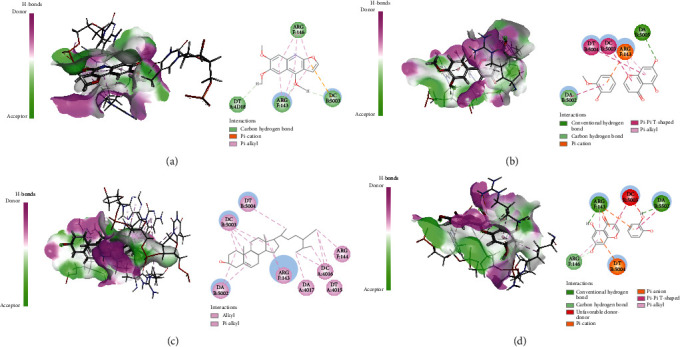
Molecular docking of JUN with 5 main ingredients: (a) kokusaginin, (b) diosmetin, (c) beta-sitosterol, and (d) quercetin, respectively.

Active ingredients and targets with high connectivity were screened out according to network pharmacology and analyzed using Discovery Studio 2019 software. The docking results of the 5 core targets and the corresponding active ingredients of TCM showed that kokusaginin, skimmianin, diosmetin, beta-sitosterol, and quercetin were main compounds of *Zanthoxylum bungeanum.* The libdock scores were higher than other components, suggesting that it is more likely to be the key drug active molecule of *Zanthoxylum bungeanum* in the treatment of DM (Figure 6, Table 3). Among them, beta-sitosterol (67.85) and quercetin (56.04) had the strong binding ability with AKT1 (Figure 7); diosmetin (79.18) and skimmianin (77.02) had the strong binding ability with IL-6 (Figure 8); diosmetin (91.09) and quercetin (107.43) had the strong binding ability with HSP90AA1 (Figure 9); beta-sitosterol (118.17) and quercetin (107.4) had the strong binding ability with FOS (Figure 10); beta-sitosterol (125.09) and diosmetin (120.99) had the strong binding ability with JUN (Figure 11). In addition, kokusaginin, skimmianin, diosmetin, beta-sitosterol, and quercetin form a hydrogen bond with the active site LYS-A: 20 (Figure 7(a)), LYS-A: 20 (Figure 7(b)), GLY-A: 16 (Figure 7(c)), ASN-A: 54 (Figure 7(d)), and THR-A: 87 (Figure 7(d)) of the protein encoded by the AKT1 gene, respectively. Diosmetin forms a hydrogen bond with the active site GLU-A: 51 and ARG-A: 168 of the protein encoded by the AKT1 gene (Figure 8(c)). Kokusaginin, skimmianin, diosmetin, and quercetin forms a hydrogen bond with the active site GLY-A: 97 (Figure 9(a)), ASN-A: 51 (Figure 9(b)), ASN-A: 106 and SER-A: 52 (Figure 9(c)), ASN-A: 51, and LEU-A: 48 (Figure 9(d)), of the protein encoded by the HSP90AA1 gene, respectively. Kokusaginin, skimmianin, diosmetin, and quercetin forms a hydrogen bond with the active site ARG-F: 146 (Figure 10(a)), ARG-F: 143 and DA-B: 5005 (Figure 10(b)), DC-B: 5003 (Figure 10(c)), and ARG-F: 143 (Figure 10(e)) of the protein encoded by FOS gene, respectively. Skimmianin and quercetin form a hydrogen bond with the active site DA-B: 5005 (Figure 11(b)), DC-B: 5002, and ARG-F: 143 (Figure 11(d)) of the protein encoded by the JUN gene, respectively.

### 3.8. Results of Cell Cytotoxicity/Viability Test

**Figure 12:**
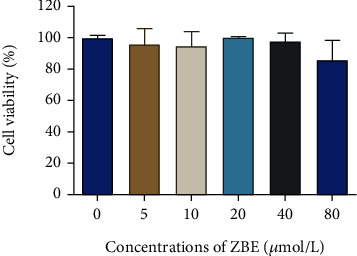
Effects of different concentrations of ZBE on HepG2 cell cytotoxicity/viability.

The cell cytotoxicity/viability test results showed that the concentration of ZBE between 5 *μ*mol/L and 80 *μ*mol/L has no toxicity to HepG2 cells. In addition, it was revealed that no significantly inhibit the proliferation of HepG2 cells when concentration of ZBE at 5 *μ*mol/L to 40 *μ*mol/L. Furthermore, ZBE exhibits the relatively stable effect on DM at concentration of 20 *μ*mol/L and 40 *μ*mol/L, respectively (Figure 12). Therefore, the 20 *μ*mol/L and 40 *μ*mol/L of ZBE were chosen for next step experiment.

### 3.9. Effects of ZBE on the Expression of AKT1, IL6, HSP90AA1, FOS, and JUN Proteins in HepG2 Cells

**Figure 13:**
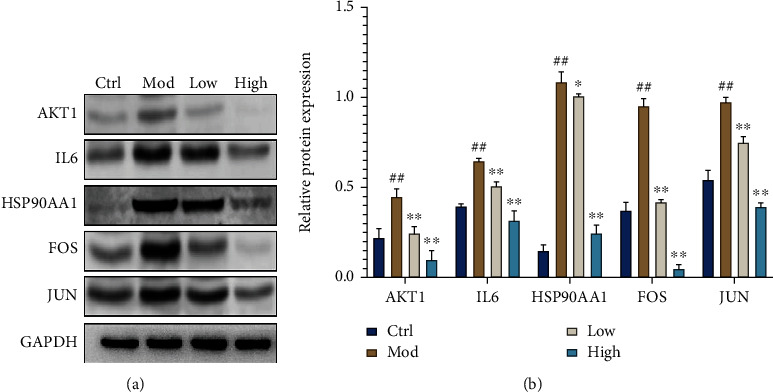
Effect of ZBE on expressions of AKT, P-AKT, FOXO1, P-FOXO1, and JNK proteins in HepG2 cells. The data represent the mean ± SD (*n* = 6). ^∗∗^*P* < 0.05 and ^∗∗^*P* < 0.01 vs. the control group; ^#^*P* < 0.05 and ^##^*P* < 0.01 vs. the model group.

Compared with the control group, the expression of AKT1, IL6, HSP90AA1, FOS, and JUN proteins in the model group was upregulated (*P* < 0.01). Compared with the model group, the protein expression in the low-concentration group (*P* < 0.05, *P* < 0.01) and the high-concentration group (*P* < 0.01) of ZBE significantly decreased. The results showed that DM could be significantly improved by downregulating the expression of related proteins (AKT1, IL6, HSP90AA1, FOS, and JUN) after being treated with different concentrations (20 *μ*mol/L and 40 *μ*mol/L) of ZBE (Figure 13).

## 4. Discussion and Conclusion

There are few studies on the treatment of diabetes-related diseases with *Zanthoxylum bungeanum* extract, and the active components, targets, and mechanisms of *Zanthoxylum bungeanum* in the treatment of DM are not completely clear. In recent years, some researchers have used network pharmacology methods and carried out systems biology, biological network construction and analysis methods to evaluate the pharmacological effects of TCM, to clarify the effective active components of TCM and potential disease targets, and then to explore the molecular mechanism of TCM to exert its efficacy [14].

In the present study, the method of network pharmacology was used to analyze and explore the active components, key targets, and signaling pathways of *Zanthoxylum bungeanum* in the treatment of DM. It was found that there were 5 active components in *Zanthoxylum bungeanum* that acted on 187 targets related to DM. The main targets to play the role of antidiabetes action are AKT1, IL6, HSP90AA1, FOS, and JUN. Using R 4.0.4 language software package and bioconductor bioinformatics software package, GO analysis and KEGG analysis were performed on the potential DM targets of *Zanthoxylum bungeanum*. The results showed that the common biological process of *Zanthoxylum bungeanum* and DM is related to positive regulation of gene expression, positive regulation of transcription, positive regulation of transcription from RNA polymerase II promoter, response to drug, positive regulation of apoptotic process, positive regulation of cell proliferation, etc. The mechanism of action of *Zanthoxylum bungeanum* in the treatment of DM involves multiple pathways including positive regulation of gene expression, positive regulation of transcription, positive regulation of transcription from RNA polymerase II promoter, response to drug, and positive regulation of apoptotic process, etc.

The main active components of *Zanthoxylum bungeanum* were kokusaginin, skimmianin, diosmetin, beta-sitosterol, and quercetin, respectively, of top 5 analyzed by network pharmacology. It was reported that kokusaginin as an anti-multidrug resistance agent in chemotherapy for breast carcinoma which is closed with AKT pathway [13]. In addition, it was reported that kokusaginine's potential as a good inhibitor against hepatitis C virus and type 2 diabetes [15, 16]. It was revealed that skimmianin treatment suppressed the transcription of TNF-a and IL-6 genes, highlighting the downregulation of cytokine production and subsequent production of PGE2, inhibited NO production, indicating its anti-inflammatory effect [17]. A previous study showed that diosmetin protects against renal injury in streptozotocin- (STZ-) induced diabetic nephropathy mice by modulating the AKT/NF-*κ*B/iNOS signaling pathway [18]. It was also found that diosmetin ameliorate type 2 diabetic mellitus by upregulating *Corynebacterium glutamicum* to regulate IRS/PI3K/AKT-mediated glucose metabolism disorder in mice [19]. Beta-sitosterol is a plant sterol which has the similar chemical structure like cholesterol [20]. Previous findings indicated that beta-sitosterol improves glycemic control through activation of IR in the adipose tissue of high-fat and sucrose-induced type-2 diabetic rats [21]. Quercetin is a polyphenol which has been shown in vitro as well as in a few animal models to have several potential anti-inflammatory as well as anticarcinogenic applications. It was revealed that quercetin exhibits anti-inflammatory properties in relation to obesity and type 2 diabetic mellitus [22]. Furthermore, it has been demonstrated that quercetin could be used to successfully prevent some of the clinical complications of diabetes [23]. A previous study also showed that quercetin intake was inversely related to the prevalence of type 2 diabetic mellitus in the Chinese population, suggesting a protective effect of quercetin in the development of type 2 diabetic mellitus [24]. Above all, all the active components of *Zanthoxylum bungeanum* exhibits various effects in the treatment of DM especially diosmetin, quercetin, and beta-sitosterol which is similar with our prediction.

AKT1 is one of 3 closely related serine/threonine protein kinases (AKT1, AKT2, and AKT3) known as AKT kinases, and it regulates many processes, including metabolism, proliferation, cell survival, growth, and angiogenesis. AKT is an important target kinase downstream of PI3K. PIP3 can bind to AKT, transfer AKT from the cytoplasm to the cell membrane, and phosphorylate Ser473 and Thr308 to fully activate AKT, thereby initiating the PI3K/AKT signaling pathway [25]. It was reported that the PI3K-AKT signaling pathway may inhibit the gluconeogenesis pathway and reduce the output of glucose, thereby exerting a hypoglycemic effect in db/db mice [26]. Previous studies have reported that *Zanthoxylum bungeanum* extract may play a hypoglycemic effect by downregulating PGC-1*α* in the liver [27]. Inflammation is closely related to the occurrence and development of diabetes. IL-6, as an important cytokine in inflammatory response, has a high content in the serum of patients with diabetes [28]. The *Zanthoxylum bungeanum* extract has an anti-inflammatory effect by inhibiting the release of TNF-*α* and IL-6 [29]. It is speculated that *Zanthoxylum bungeanum* may play a hypoglycemic effect by inhibiting the inflammatory response. The pathways involved in HSP90AA1 include BH4 synthesis, recycling, recovery, and regulation, eNOS activation, and VEGFR2-mediated vascular permeability [30], consistent with the pathways involved in the treatment of diabetes by *Zanthoxylum bungeanum.* As a class of nuclear protein transcription factors, FOS proteins play an important role in regulating cell growth, division, proliferation, and programmed death [31]. It was reported that the reduced expression of the immediate early gene FOS as an indicator of pain transmission supports the diabetes-induced loss of sensation in this type 1 model of diabetes [32]. C-JUN protein is one of the activating protein-1 (AP-1) transcription complexes and is the most transcriptionally active transcription factor in the AP-1 complex. C-JUN protein expression and activation can be stimulated, thereby regulating biological processes such as cell proliferation and apoptosis [33]. The inflammatory kinase JNK is proposed to be a key player, as whole-body JNK1-null mice protects against obesity-induced insulin resistance. This presents a potential therapeutic target (JUN) to restore insulin sensitivity and preserve *β*-cell function [34].

Taken together, the active components of *Zanthoxylum bungeanum* mainly including kokusaginin, skimmianin, diosmetin, beta-sitosterol, and quercetin. The findings indicated that AKT1, IL6, HSP90AA1, FOS, and JUN are the most likely targets for *Zanthoxylum bungeanum* on treating DM. Experiment verification results revealed that DM could be significantly improved by downregulating the expression of AKT1, IL6, HSP90AA1, FOS, and JUN proteins after administrated 20 *μ*mol/L and 40 *μ*mol/L of ZBE. *Zanthoxylum bungeanum* is an effective drug in treatment of DM related to above core targets. The present study preliminarily clarifies that *Zanthoxylum bungeanum* may treat DM through multitargets and multipathways, which provides a new idea and points out a new direction for further experimental research and clinical application.

## Figures and Tables

**Table 1:** General information of active ingredients of *Zanthoxylum bungeanum*.

Mol ID	Molecule name	MW	OB (%)	DL	HL
MOL013271	Kokusaginin	259.28	66.68	0.2	-3.19
MOL002663	Skimmianin	259.28	40.14	0.2	-2.43
MOL002881	Diosmetin	300.28	31.14	0.27	16.34
MOL000358	Beta-sitosterol	414.79	36.91	0.75	5.36
MOL000098	Quercetin	302.25	46.43	0.28	14.4

**Table 2:** Topological parameters of core target genes in the intersection genes of *Zanthoxylum bungeanum* and diabetes.

Core target gene	Betweenness centrality	Closeness centrality	Degree	Score
JUN	0.10115515	0.40967742	45	929.6329377
HSP90AA1	0.05450466	0.39379845	40	1040.573485
AKT1	0.13233367	0.41983471	40	1933.09329
FOS	0.01853274	0.36285714	28	1026.474052
IL6	0.01959522	0.34002677	23	1484.812682

**Table 3:** The results of molecular docking.

Compound	Target	PDB	Libdock score
Kokusaginin	AKT1	1 h10	40.36
Skimmianin	AKT1	1 h10	26.46
Diosmetin	AKT1	1 h10	56.65
Beta-sitosterol	AKT1	1 h10	67.85
Quercetin	AKT1	1 h10	56.04
Kokusaginin	IL6	1alu	66.33
Skimmianin	IL6	1alu	77.02
Diosmetin	IL6	1alu	79.18
Beta-sitosterol	IL6	1alu	70.68
Quercetin	IL6	1alu	71.44
Kokusaginin	HSP90AA1	1byq	88.68
Skimmianin	HSP90AA1	1byq	73.36
Diosmetin	HSP90AA1	1byq	91.09
Beta-sitosterol	HSP90AA1	1byq	0
Quercetin	HSP90AA1	1byq	89.96
Kokusaginin	FOS	1a02	93.34
Skimmianin	FOS	1a02	87.24
Diosmetin	FOS	1a02	105.47
Beta-sitosterol	FOS	1a02	118.17
Quercetin	FOS	1a02	107.43
Kokusaginin	JUN	1jnm	98.24
Skimmianin	JUN	1jnm	0
Diosmetin	JUN	1jnm	120.99
Beta-sitosterol	JUN	1jnm	125.09
Quercetin	JUN	1jnm	104.87

## Data Availability

All data included or relevant to the study are available upon request by contact with the corresponding author.
